# To Vaccinate or Not to Vaccinate—This Is the Question among Swiss University Students

**DOI:** 10.3390/ijerph18179210

**Published:** 2021-08-31

**Authors:** Julia Dratva, Aylin Wagner, Annina Zysset, Thomas Volken

**Affiliations:** 1 Institute of Health Sciences, ZHAW Zurich University of Applied Sciences, Katharina-Sulzer-Platz 9, 8401 Winterthur, Switzerland; aylin.wagner@zhaw.ch (A.W.); annina.zysset@zhaw.ch (A.Z.); thomas.volken@zhaw.ch (T.V.); 2 Medical Faculty, University of Basel, Klingelbergstrasse 61, 4056 Basel, Switzerland

**Keywords:** vaccination intention, vaccine hesitancy, students, COVID-19 vaccine, SARS-CoV-2 vaccine, young adults

## Abstract

The speed and innovation of the COVID-19 vaccine development has been accompanied by insecurity and skepticism. Young adults’ attitude to vaccination remains under investigation, although herd immunity cannot be reached without them. The HEalth in Students during the Corona pandemic study (HES-C) provided the opportunity to investigate vaccination intention in 1478 students in the sixth survey wave (January 2021), including vaccination intention, psychological antecedents of vaccine hesitancy, trust in government’s vaccination strategy, and vaccination history. Associations with vaccination intention were analyzed with multivariate ordinal regression and predicted margins were calculated adjusting for gender, age, anxiety, health profession, and subjective health status. A third was decided (yes 25.1%, no 7.6%), and 68% were unsure about getting the COVID-19 vaccine when available. Next to demographic characteristics, vaccination history (influenza vaccination OR = 1.39; 95% CI: 1.06–1.83, travel vaccination OR = 1.29; 95% CI: 1.04–1.60), trust in vaccination strategy (OR = 2.40; 95% CI: 1.89–3.05), and 5C dimensions were associated with vaccination intention: confidence (OR = 2.52; 95% CI: 2.09–3.03), complacency (OR = 0.79; 95% CI: 0.66–0.96), calculation (OR = 0.79; 95% CI: 0.70–0.89), constraints (OR = 1.18; 95% CI: 0.99–1.41), and collective responsibility (OR = 4.47; 95% CI: 3.69–5.40). Addressing psychological antecedents and strengthening trust in official strategies through targeted campaigns and interventions may increase decisiveness and result in higher vaccination rates.

## 1. Introduction

The SARS-CoV-2 vaccination is as unprecedented as the COVID-19 pandemic itself. Never before has a vaccination been developed so quickly after the identification of a virus. This success was possible due to an international effort and a new methodology, messenger RNA, being applied in the development of the vaccine [1,2]. This quick development phase has been coupled with the insecurities and skepticism of the public [3], on one hand related to the methodology involving genetic mechanisms and until now non-existent evidence on long-term effects, and on the other hand to pre-existing vaccine hesitancy.

Vaccine hesitancy refers to a delay in acceptance or refusal of vaccination, despite the availability of vaccination services. It is complex, context specific, and variable across time, place, and vaccine [4]. Vaccine hesitancy is present in the majority of WHO member states [5]. The top three cited reasons for vaccine hesitancy globally are risk-benefit (scientific evidence), lack of knowledge and awareness of vaccination and its importance, and socio-cultural reasons such as religion, culture, gender, and socioeconomic issues regarding vaccines [5]. 

While vaccine hesitancy has been extensively studied in the general adult population, and in particular parents, vaccine hesitancy in young adults has been under-investigated as this age group was not in the focus of public health vaccine strategy and communication. However, the current COVID-19 pandemic also brings the younger population into the spotlight of vaccine strategies. COVID-19 vaccine hesitancy poses significant risks both to people who delay or refuse vaccination and to the wider community. It prevents the achievement of the thresholds necessary for herd immunity, unnecessarily perpetuating the pandemic [6]. First studies indicate that vaccine hesitancy is higher in young adults [7]. With respect to university students, they are in particular vulnerable to COVID-19 infection due to shared housing, the reopening of college campuses and activities, and the necessity to travel between their home and campus [8]. Furthermore, college campuses have the potential to become “superspreaders”. Such outbreaks tend to spread into the neighboring communities, driving the pandemic [9]. Thus, it is critical to address COVID-19 vaccine hesitancy in students for better controlling COVID-19 spread. 

Only recently, several studies have been published aimed at gaining a deeper understanding of COVID-19 vaccine hesitancy and willingness in students. In a study in the U.S., almost half (47.5%) of participants reported hesitancy regarding the COVID-19 vaccination [8]. Non-hesitant college students in the U.S. showed a higher behavioral confidence (i.e., being sure of properly following through with COVID-19 vaccination despite obstacles), participatory dialogue, and changes in the physical environment (i.e., whereby the person has necessary resources for performing a given behavior) than students who were vaccine hesitant [8]. Sallam et al. [10] investigated COVID-19 vaccine acceptance among university students in Jordan and found a low intention to get vaccinated against COVID-19 (34.9%). Higher rates were observed among males (42.1%) and students at Health Schools (43.5%) as compared to other faculties. A major result of this study was the independent correlation between the belief in conspiracy and COVID-19 vaccine hesitancy [10]. 

Switzerland introduced containment measures including closure of schools and universities mid-March 2020 (17 March 2020) with the beginning of the first wave. As in most European countries, the lockdown caused case numbers to drop, resulting in more relaxed containment measures over the summer and a resurgence in COVID-19 cases and deaths from September 2020 to January 2021 (second wave). The stringency of the containment measures was adapted accordingly (Supplementary Figure S1). 

In Switzerland, COVID-19 vaccine hesitancy has been rarely studied. Only recently, the first data regarding COVID-19 vaccine hesitancy in the general population have been published [11]. They show that the differences between the age groups are particularly striking. There are comparatively many people under 35 who are unwilling to be vaccinated, and whose confidence in vaccination, the responsible authorities, and the pharmaceutical companies is lower than in all other age groups. Interestingly, while the youngest age group of 15–24 year olds shows equally high vaccine hesitancy, it is not accompanied by a particular skepticism towards the vaccination and its context. The perception of being less affected by COVID-19 and less at risk may be an explanation [11].

To date, little is known on Swiss students’ attitudes towards vaccination in general and specifically regarding COVID-19, nor about differences across faculties and professions. Understanding the factors related to COVID-19 vaccine hesitancy and willingness in university students is critical for evidence-based public health communication and efforts to increase vaccination rates [12]. The HEalth in Students during the Corona pandemic (HES-C) study [13] provided the opportunity to investigate vaccine willingness, hesitancy, and predictors in this under-investigated age group.

## 2. Materials and Methods

### 2.1. Survey Design and Study Design

Study data stem from the HES-C study [13], which aims to (1) evaluate the health of students during the pandemic, (2) investigate changes in health behavior and associated factors, and (3) assess students’ perception of the pandemic and related measures and their impact on students’ lives. All enrolled students at the Zurich University of Applied Sciences (ZHAW) (*N* = 13,500) were invited to participate in eight consecutive surveys administered between April 2020 and January 2021 in an open-cohort design. Each survey lasted about 20–25 min and ran for a total period of seven working days. Participants’ informed consent was obtained before each survey. In the present study, we used cross-sectional data from the sixth survey wave (*N* = 1478). Data collection of the sixth wave took place from 21 to 29 January 2021. The study was approved by both the local cantonal ethics committee (BASEC-Nr. Req-2020–00366) and the ZHAW data protection officer.

### 2.2. Missing Data

Of the 1478 students who participated in the study, i.e., started the survey, 1358 (91.9%) completed the survey. Information on COVID-19 vaccination intention, our primary outcome, was provided by 1357 students and complete data for all variables used in the analyses was available for 1297 students. Missing values were most prevalent in the variable “trust in the Federal Council’s vaccination strategy” (2.3%, *n* = 31) and ranged from 0% to 0.7% for the remaining variables. Incomplete cases amounted to 4.4% of the data. Moreover, we used the Stata *mvpatterns* and *misschk* commands to cross-tabulate and plot all combinations of missing and non-missing values of variables used in the analyses. Using visual inspection, we detected no systematic patterns in the missing data. For the above reasons, we did not impute missing data [14] and included 1297 complete cases throughout all analyses.

### 2.3. Measures

#### 2.3.1. Outcome: Vaccination Intention

COVID-19 vaccination intention was measured with a single item, used in the Swiss national Corona Immunitas Study [15], with a 6-point Likert scale: “As soon as a COVID-19 vaccine is available, do you think you will get vaccinated?” (1 = no, 2 = probably no, 3 = undecided, 4 = yes, after others, 5 = probably yes, 6 = yes). 

#### 2.3.2. Predictors

The psychological antecedents of vaccination were measured using the validated 5C scale [16]. The 5C scale is a self-assessment questionnaire that measures 5 different psychological dimensions of vaccine hesitancy, i.e., confidence in vaccines (confidence), complacency (perceived disease risk), constraints (perceived barriers to vaccination), calculation (information-seeking behavior), and collective responsibility (awareness of social benefits of vaccination). The questionnaire is comprised of 15 items to be rated on a 5-point Likert scale (from “Strongly disagree” to “Strongly agree”). Scores on each sub-scale represent the mean scores of the scale’s respective items and range from 1 to 5, with higher values representing more agreement. 

Students were asked to indicate their degree of trust in the government’s vaccination strategy on a 4-point Likert scale, i.e., “Would you say that you have no trust, little trust, trust or complete trust in the Federal Council’s vaccination strategy?”. Responses were then dichotomized as 0 (i.e., no trust, little trust) and 1 (i.e., trust, complete trust). With respect to previous vaccination experience, students were asked whether they had ever been vaccinated against seasonal influenza (0 = no, 1 = yes) and for travel medical precautions (0 = no, 1 = yes), e.g., yellow fever.

#### 2.3.3. Covariates

Socio-demographic covariates included students’ age at the last birthday in complete years, gender (0 = women, 1 = men), and faculty (0 = all other faculties, 1 = department of health professions). Wellbeing-related covariates included students’ self-rated health and anxiety. With respect to self-rated health, participants were asked “In general, would you say that your health is very poor, poor, fair, good, very good?”. Due to the low frequency of students who indicated very poor or poor health, i.e., 5.7% (*n* = 66), we dichotomized students into those with very good or good health and those with fair, poor, or very poor health with the latter category being the reference category. Anxiety was measured with the General Anxiety Disorder Scale-7 (GAD-7) [17]. The GAD-7 is a self-assessment questionnaire that measures the level of perceived anxiety in the last two weeks. The questionnaire is comprised of 7 items to be rated on a 4-point Likert scale (from “not at all” to “nearly every day”). The resulting sum score ranges from 0 to 21, with lower values indicating a lower level of anxiety, and is categorized into four severity levels of anxiety: minimal (0–4), mild (5–9), moderate (10–14), and severe (15–21).

### 2.4. Statistical Analyses

Descriptive statistics (i.e., frequencies, percent, median, interquartile range, mean, standard deviation) were applied to evaluate the characteristics of the samples. We used univariate Student’s t-tests for continuous variables and Chi-square-tests for categorical variables to assess univariate group differences. Ordinal logistic regression models, i.e., proportional odds models, were used to estimate adjusted vaccination intention. Adjustment comprised age, gender, faculty, health status, generalized anxiety, trust in the Swiss Federal Council’s vaccination strategy, previous seasonal influenza vaccination, previous travel vaccination, and psychological vaccination antecedents (confidence, complacency, constraints, calculation, collective responsibility). We report odds ratios (OR) with corresponding 95% confidence intervals (95% CI), predictive margins (average predicted probability), and average marginal effects for major predictors. For the ordinal logistic model, the underlying proportional odds assumptions were checked using Brant test [18] and met in all models. Statistical significance was established at *p* < 0.05. We used Stata Version 15.1 (StataCorp, College Station, TX, USA) for statistical analyses.

## 3. Results

### 3.1. Participants’ Characteristics

The analytic sample consists of 912 women (70.3%) and 385 men (29.7%) (Table 1). Overall, the median age of students was 24 years; the interquartile range (IQR) was 22–27 years. The corresponding age for female and male students was 24 years (IQR: 22–27) and 24 years (IQR: 23–27) with no substantial differences in age between gender (*p* = 0.622). The percentage of health profession students was 23.1%, which is considerably higher than their share in the total ZHAW student population (13.0%). More women than men were health profession students (*p* ≤ 0.001). Roughly, three quarters of the students reported their health to be very good or good, and women assessed their subjective health slightly better than men did (*p* = 0.007). The prevalence of moderate to severe anxiety levels was 33.5% with no substantial differences between female and male students (*p* = 0.765). 

A quarter of the students indicated that they would get vaccinated against COVID-19 (yes 25.1%), and 7.6% responded they would not get vaccinated. Two-thirds were not absolutely sure (probably yes 24.1%, probably no 13.9%), undecided (14.4%), or wished to wait for others to get vaccinated first (15.0%). Positive vaccination intention in women was slightly lower than in men (*p* = 0.065). Trust in the Swiss Federal Council’s vaccination strategy was held by 45.2% of women and 51.7% of men (*p* = 0.032). A fifth of female (21.2%) and male students (18.7%) had previously been vaccinated against seasonal influenza at least once (*p* = 0.315), while about half of the students (49.2%, respectively 40.5%), reported having had a travel vaccination in the past (*p* = 0.004). With respect to the psychological antecedents of vaccination [16], female as compared to male students expressed lower levels of confidence (*p* = 0.003), complacency (*p* = 0.010), and constraints (*p* = 0.064; borderline significant), and higher levels of calculation (*p* < 0.001) and collective responsibility (*p* = 0.088; borderline significant).

### 3.2. Factors Associated with COVID-19 Vaccination Intention

Adjusting for all other covariates (Table 2, crude OR Supplementary Table S1), vaccination intention was higher in participants who expressed trust in the Swiss Federal Council’s vaccination strategy (OR = 2.40; 95% CI: 1.89–3.05). Similarly, vaccination intention was higher in students who reported having been vaccinated at least once against influenza (OR = 1.39; 95% CI: 1.06–1.83) or for travel medical precautions (OR = 1.29; 95% CI: 1.04–1.60). With respect to the 5C vaccination antecedents, students who expressed more confidence in vaccination were more likely to consider being vaccinated (OR = 2.52; 95% CI: 2.09–3.03) while higher complacency, i.e., not perceiving disease as high risk, was associated with lower vaccination intention (OR = 0.79; 95% CI: 0.66–0.96). Engaging in extensive information seeking, captured by the calculation dimension, was associated with lower vaccination intention (OR = 0.79; 95% CI: 0.70–0.89). Students who were more prone to consider aspects pertaining to collective responsibility, i.e., willingness to protect others, were more likely to consider being vaccinated (OR = 4.47; 95% CI: 3.69–5.40). The dimension constraints, e.g., perceived geographical or financial barriers, were positively associated with vaccination intention but were only borderline significant (OR = 1.18; 95% CI: 0.99–1.41). 

With respect to student characteristics, included in the model to control for potential confounding, some of the differences seen in univariate analyses remain significant. Male students (OR = 1.30; 95% CI: 1.01–1.66) and older students (OR = 1.03; 95% CI: 1.01–1.05) were more likely to consider COVID-19 vaccination, and very good or good health was negatively associated with vaccination intention (OR = 0.74; 95% CI: 0.57–0.96). Health profession students compared to all other students were no longer significantly different in their vaccination intention. Additionally, different levels of anxiety were not associated with vaccination intention. 

The predictive margins for each of the vaccination intention categories (no; probably no; undecided; yes, after others; probably yes; yes) were 0.076 (95% CI: 0.065–0.086), 0.135 (95% CI: 0.119–0.151), 0.144 (95% CI: 0.0.127–0.162), 0.151 (95% CI: 0.132–0.169), 0.243 (95% CI: 0.221–0.264), and 0.251 (95% CI: 0.232–0.270), respectively. These average predicted probabilities lie near the empirical distribution of vaccination intention presented in Table 1. 

**Figure 1:**
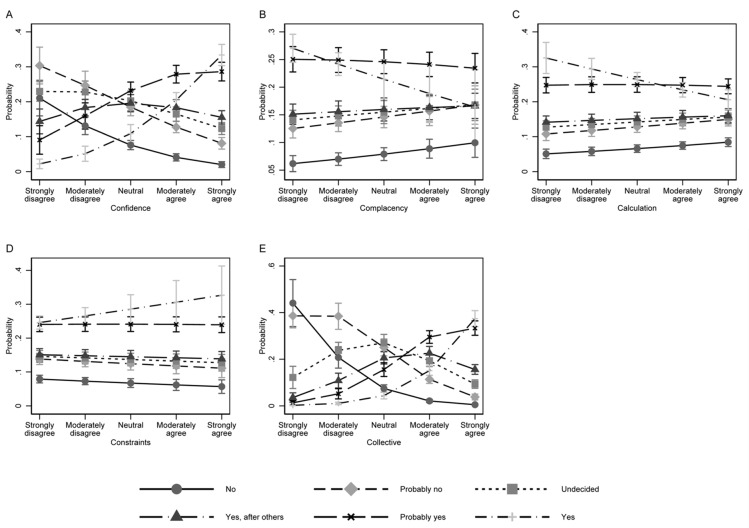
Predictive margins of vaccination intention categories by 5C antecedents. Legend: Average predicted probability of each vaccination intention category: confidence (**A**), complacency (**B**), calculation (**C**), constraints (**D**), and collective (**E**). Whiskers show the 95% confidence interval of the point estimates.

**Figure 2:**
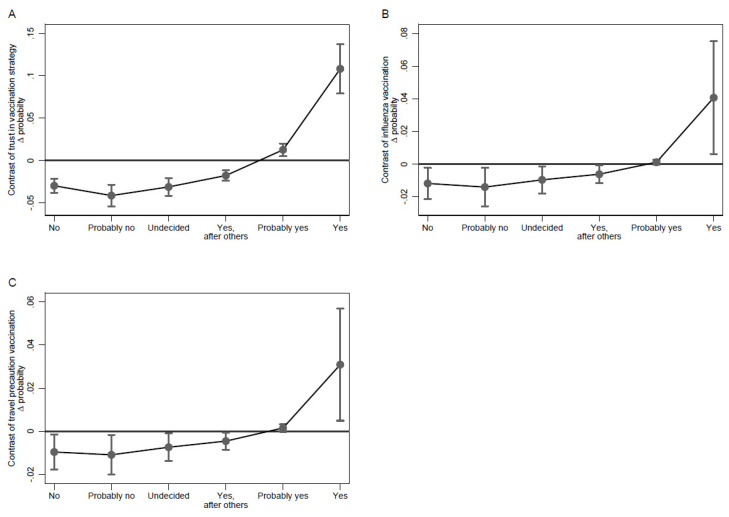
Average marginal effect of trust in the government’s vaccination strategy, seasonal influenza vaccination, and travel vaccination. Legend: Difference in probabilities by vaccination intention category for participants who trust the vaccination strategy (**A**), who had an influenza vaccination (**B**), who had a travel precaution vaccination (**C**). Whiskers show the 95% confidence interval of the point estimates.

Figure 1 depicts predicted probabilities of each vaccination intention category over the range of the different 5C antecedents. Vaccination intention trajectories were most pronounced for the confidence and collective dimensions (Figure 1A,E). Based on the ordinal logistic regression model, the vaccination intention categories “yes” or “probably yes” were more likely in students who expressed strong confidence in vaccination (strongly agree), while students who had little confidence in vaccination (strongly disagree) were more probable to report the vaccination intention categories “no”, “probably no”, or “undecided”. Along the scale, the probability of the latter vaccination intention categories coherently decreased or increased, respectively. A similar pattern can be observed for the collective dimension where after adjustment the “yes” or “probably yes” vaccination intention category was most likely for students who strongly agreed that vaccination is a collective endeavor while those who strongly disagreed were more likely to be in the “no” and “probably no” categories. For the remaining three dimensions, the patterns were less coherent. While students who strongly disagreed on the complacency scale were most likely to be to belong to the “yes” or “probably yes” group, there was no pronounced decreasing trend over the complacency scale (Figure 1B). Moreover, students who strongly agreed on the complacency scale were still more likely to report “probably yes” or equally likely “yes”, “yes, after others”, “undecided”, or “probably no” regarding their vaccination intention. A similar pattern can be observed for the calculation and constraints dimension. These findings are in line with the fact that the average marginal effects of confidence and collective responsibility on the probability, i.e., up to 0.18, far exceeded those of the remaining 5C dimensions where effects on probability were in a negligible range of ±0.02. With respect to effects on probability, trust in the Federal Council’s vaccination strategy and influenza vaccination too contributed substantially, especially regarding the “yes” vaccination intention category (Figure 2). To a lesser degree, this is also the case for travel precaution vaccination. 

## 4. Discussion

In our sample of Swiss students, a considerable proportion of young people were unsure or undecided regarding their vaccination intention. Only a third had made up their mind in January 2020. Overall, half responded they would or probably would get vaccinated against COVID-19. This rate is somewhat lower compared to rates reported for other countries [12,19]. Several demographic characteristics, vaccination history, trust in the government’s vaccination strategy, and psychological vaccination antecedents were positively associated with COVID-19 vaccination intention.

An important finding of this study is that four of five specific dimensions of the generalized measure of psychological vaccination antecedents, the 5C scale [16], were significantly associated with COVID-19 vaccination intention among Swiss students. This is the first study, to our knowledge, using the 5C scale in students. Higher scores on *confidence* and *collective responsibility* were associated with a positive COVID-19 vaccination intention, while lower scores on *complacency* and *calculation* were associated with a negative intention. This is consistent with similar findings by Kwok et al. [20] among health care workers in Hongkong and Dorman et al. [21] in a large convenience sample in the US, where the strongest correlations and effects were found for confidence in the safety of the vaccine and concern about protecting others through vaccination (collective responsibility) [21]. The dimension *constraints* was not significantly associated with vaccination intention.

The 5C antecedent *confidence* relates to trust in the safety and effectiveness of the COVID-19 vaccine as well the belief that public authorities decide in the best interest of the community. While trust in public authorities is included in the measure, apparently it did not capture all aspects of trust, as the predictor “*trust in the government’s vaccination strategy*” proved to be an independent factor and was significantly and positively associated with vaccination intention. Trust in the government is generally high in Switzerland [22], but with regard to COVID-19 it decreased over the course of the pandemic [23]. For example, confidence in the government’s competence to cope with the COVID-19 pandemic was almost double in the ZHAW student body in the first survey in April 2020, with 87% voicing trust in the federal council and 97% in the federal office of public health, as compared to the current confidence in the government’s vaccination strategy [24]. Strengthening confidence and trust in public authorities seems to be an essential factor to increase COVID-19 vaccination rates, as confirmed by other studies [7,25]. For example, Murphy et al. [7] observed that COVID-19 vaccine-hesitant or -resistant adults in Ireland and the UK distrust experts and authority figures (i.e., scientists, health professionals, the state) more and present more conspiratorial and paranoid beliefs reflecting a lack of trust in the intentions of others. We further investigated the *vaccination history*, under the assumption that previous vaccination decisions are likely to predict future decisions and are closely related to the dimension of confidence. Previous vaccinations may indicate a general trust in vaccines and an understanding of the health benefit of vaccinations. Our results yield that, in fact, students who reported a previous seasonal influenza vaccination or travel vaccination had an increased odds of positive COVID-19 vaccination intention. Schwarzinger et al. [26], investigating French 16–64 year-olds, also showed that both outright vaccine refusal and vaccine hesitancy were significantly associated with poor compliance with recommended vaccinations in the past, and Ruiz and Bell [27] observed that influenza vaccine uptake was a significant predictor of vaccination intent in the general American public.

Furthermore, *collective responsibility*, defined as the willingness to protect others by the means of one’s own vaccination, was observed to be an important factor in COVID-19 vaccination intention in this study. While this result is also found in other populations, such as health professionals [20,28] and older populations [29,30], it may have been underestimated in younger individuals. In our study, students who rated high on collective responsibility showed five-times higher odds of getting vaccinated than students who rated lower. To tackle COVID-19 vaccine hesitancy, increasing collective responsibility in young adults seems to be critical.

*Complacency*, i.e., a low risk perception, is a significant predictor of COVID-19 vaccination intention. This is consistent with Schwarzinger et al. [26] as well as Ruiz and Bell [27] who highlighted that vaccine refusal was associated with a lower perceived severity of COVID-19. In particular, younger people are more likely to be complacent about the risk of COVID-19 than older people [24] because they are less likely to be hospitalized or to die from COVID-19 [31]. Indeed, initially, the communication about COVID-19 nurtured the idea that young people had little to fear [32]. In our study, we find the expected direction of effect; however, it is overall a small effect. Possibly, with increasing knowledge on the short- and long-term health risks, such as long COVID [33,34], and the personal inconveniences resulting from a suspected or true infection, such as isolation or quarantine, the perception of risk has changed.

The dimension *calculation*, referring to the correlation between individuals’ engagement in extensive information-seeking behavior and decision making, had a significant influence on COVID-19 vaccination intention, but again only a small overall effect. The underlying assumption is that extensive information seeking leads to a higher exposure to vaccine-critical individuals, since critical voices are disproportionally more prominent in the internet [16], and media controversies and vaccine-critical sources have a negative impact on vaccine willingness [35,36,37,38,39,40,41]. The information source “social media” has been found to be associated with COVID-19 conspiracy beliefs and with higher vaccine hesitancy [42]. While international studies indicate that students often rely on social media information [40,43], we do not observe this behavior in our student body. A previous published paper found that ZHAW students’ first and second choice of information source in relation to COVID-19 were public media and public health institutions and social media was only the third most frequent information source [24]. This may partially explain the small effect of calculation, next to the fact that students most likely have a higher data literacy than the general population.

While our main predictors were the psychological antecedents, trust in vaccination strategy, and previous vaccination history, it is worthwhile to report the findings on socio-demographic covariates and health status. Most COVID-19 vaccination literature reports a *gender* difference in vaccine hesitancy [7,25,26,27,44]; a recent rapid review, however, indicated a large inconsistency of results [45]. In our study, male participants were more willing to be vaccinated than females, independent of psychological antecedents, vaccination history or trust in vaccination strategy. Furthermore, higher *age* is known to be associated with lower vaccine hesitancy or refusal, and this association is supported by recent COVID-19 publications [11,25,27]. The general explanation for this age effect is an age-dependent perception of the individual risk. Albeit the small age range covered by our study participants, we observed a small but significant positive age effect. However, the student population does not fall into the *at-risk* age group; therefore, we must assume other factors underlying the age effect. One explanation might be that older students tend to have older parents for whose health they are concerned. In fact, in an earlier analysis [24], we identified that the concern expressed for parents was higher than concern for students’ own health. In agreement with most literature, we found students with better self-reported *health status* to have a lower intention to get vaccinated. For example, Schwarzinger et al. [26] indicated that absence of chronic conditions is associated with vaccine hesitancy. Ruiz and Bell [27] observed that intent to vaccinate was highest for people with pre-existing medical conditions. However, the literature is inconsistent, as Kelly et al. [44] reported that individuals with underlying medical conditions or morbid obesity were no more willing to get vaccinated against COVID-19 than their lower risk counterparts. 

Our study investigated vaccine hesitancy prior to vaccination being available for this age group; however, the vaccines themselves were approved for the respective age range and it was only a matter of time that COVID-19 vaccines would become available. The survey was held in January 2021, in a phase of highly stringent containment measures and near the end of the second wave. Therefore, the COVID-19 pandemic was very present and heavily discussed in the media. Students experienced themselves many containment measures, from online teaching, masks in public places and transport, or restrictions in movement and meeting family and friends. One might have expected a high understanding of the importance of vaccinations over the course of the COVID-19 pandemic at the given time. Nevertheless, the hypothetical wording may have influenced the response, and in this early stage of vaccination campaigns students may not have made up their mind. We captured this indecisiveness with the answer option “probably yes *or* no”. The ordinal logistic regression analyses allowed us take various steps of indecisiveness into account, which would have been lost in dichotomizing the variable responses. 

A critical point is the representativity of our findings. For one, more women participated in the survey, which might have led to an overestimation of vaccine hesitancy in the crude data. However, the main analysis, the ordinal logistic regression, adjusts for gender. Secondly, the sample consisted of students from a German-speaking Swiss university of applied sciences and was representative of the student body. While we are confident that the study can be generalized to other German-speaking universities, it should not be generalized to French- or Italian-speaking language regions in Switzerland, especially since vaccine hesitancy seems to be higher in Italian or French speakers [11]. Further, the sample is not representative for young adults in general. While Swiss universities of applied science attract students from different educational paths and backgrounds, they belong to the more highly educated population. Several studies reported that individuals with a high education level were more likely to be vaccinated against COVID-19 than individuals with a low education level [10,45,46,47,48,49,50]. 

## 5. Conclusions

Addressing psychological antecedents of vaccination intention in communications and interventions may result in more targeted and successful campaigns. Our results provide valuable evidence for future public health policy and vaccination campaigns. For one, the data show that students are highly susceptible to the collective dimension of vaccination. Second, addressing previous vaccination choices such as travel or influenza vaccinations may be a good angle to address hesitancy. Third, low risk perception predicts low vaccination intention and, thus, needs to be addressed in health communications, and further, strengthening and reassuring the confidence in the vaccination strategy and vaccination itself must be a central aim in vaccination campaigns. Universities themselves provide a good setting to reach students and to launch information campaigns. Additionally, lastly, the repeatedly observed difference by gender needs further investigations to better target the more hesitant female gender.

## Figures and Tables

**Table 1:** Participant characteristics.

Variable	Statistics	Women		Men		Total	
Gender	*n* (%)	912	(70.3)	385	(29.7)	1297	(100.0)
Age	Md (IQR)	24	(22–27)	24	(23–27)	24	(22–27)
Health professions student ***	*n* (%)	279	(30.6)	21	(5.5)	300	(23.1)
(Very) good health status **	*n* (%)	689	(75.6)	263	(68.3)	952	(73.4)
Anxiety level	*n* (%)						
*Minimal (0*–*4)*		246	(27.0)	109	(28.3)	355	(27.4)
*Mild (5*–*9)*		358	(39.3)	149	(38.7)	507	(39.1)
*Moderate (10*–*14)*		211	(23.1)	81	(21.0)	292	(22.5)
*Severe (15*–*21)*		97	(10.6)	46	(12.0)	143	(11.0)
Trust in vaccination strategy **	*n* (%)	412	(45.2)	199	(51.7)	611	(47.1)
Previous vaccinations							
Seasonal influenza vaccination	*n* (%)	193	(21.2)	72	(18.7)	265	(20.4)
Travel vaccination *	*n* (%)	449	(49.2)	156	(40.5)	605	(46.7)
5C vaccination antecedents	Mean (SD)						
Confidence **		3.88	(0.87)	4.04	(0.89)	3.93	(0.88)
Complacency *		1.96	(0.71)	2.07	(0.82)	1.99	(0.75)
*Constraints*		1.36	(0.60)	1.43	(0.71)	1.38	(0.64)
Calculation ***		3.73	(0.90)	3.44	(1.05)	3.64	(0.96)
*Collective responsibility*		4.04	(0.91)	3.94	(1.04)	4.01	(0.95)
COVID-19 vaccination intention	*n* (%)						
*No*		71	(7.8)	27	(7.0)	98	(7.6)
*Probably no*		126	(13.8)	54	(14.0)	180	(13.9)
*Undecided*		144	(15.8)	43	(11.2)	187	(14.4)
*Yes, after others*		136	(14.9)	58	(15.1)	194	(15.0)
*Probably yes*		226	(24.8)	87	(22.6)	313	(24.1)
*Yes*		209	(22.9)	116	(30.1)	325	(25.1)

*n* = number of observations; Md = median; IQR = interquartile range; SD = standard deviation; difference across gender: *** *p* < 0.001, ** *p* < 0.01, * *p* < 0.05. We used univariate Student’s t-tests for continuous variables and Chi-square-tests for categorical variables to assess univariate group differences.

**Table 2:** COVID-19 vaccination intention—adjusted ordinal logistic regression model.

Variable	OR	*p*	95% CI
Gender (ref = women)			
*Men*	1.30	0.038	1.01–1.66
Age (years)	1.03	0.001	1.01–1.05
Health professions student (ref = no)			
*Yes*	0.94	0.621	0.72–1.22
Health status (ref = poor-fair health)			
*Very good/good health*	0.74	0.026	0.57–0.96
Generalized anxiety (ref = minimal (0–4))			
*Mild (5*–*9)*	0.94	0.663	0.73–1.23
*Moderate (10*–*14)*	0.97	0.854	0.71–1.33
*Severe (15*–*21)*	0.73	0.136	0.48–1.11
Trust in vaccination strategy (ref = no)			
*Yes*	2.40	0.000	1.89–3.05
Seasonal influenza vaccination (ref = no)			
*Yes*	1.39	0.019	1.06–1.83
Travel vaccination (ref = no)			
*Yes*	1.29	0.020	1.04–1.60
5C vaccination antecedents			
*Confidence*	2.52	0.000	2.09–3.03
*Complacency*	0.79	0.018	0.66–0.96
*Constraints*	1.18	0.072	0.99–1.41
*Calculation*	0.79	0.000	0.70–0.89
*Collective responsibility*	4.47	0.000	3.69–5.40
Cut points			
*#1*	4.13		2.89–5.36
*#2*	6.44		5.17–7.71
*#3*	7.96		6.66–9.26
*#4*	9.23		7.91–10.55
*#5*	11.12		9.77–12.48
Number of observations	1297		
Pseudo R-squared	0.29		
Likelihood ratio Chi2 (15)	1308		
*p* > Chi2	0.000		

ref = reference category; OR = odds ratio; *p* = probability; 95% CI = 95% confidence interval; cut points mark boundaries on the latent variable y where the outcome changes, i.e., the probability of an observed outcome for a given x is the area under the curve between a pair of cut points.

## Data Availability

Data have not been made publicly available so far since the HES-C data collection was terminated only in June 2021. The study data presented in this study are available on request from the corresponding author.

## Supplementary Materials

The following are available online at https://www.mdpi.com/article/10.3390/ijerph18179210/s1, Figure S1: Epidemiological data and KOF Stringency Index over the duration of the HES-C study, Table S1: COVID-19 vaccination intention—unadjusted ordinal logistic regression model.
