# Supplementary material for: To Vaccinate or Not to Vaccinate—This Is the Question among Swiss University Students
Source: Int J Environ Res Public Health. 2021 Aug 31;18(17):9210. doi: 10.3390/ijerph18179210 (PMC8431304; doi:10.3390/ijerph18179210)
Supplement: Supplementary file 1 [file ijerph-18-09210-s001.zip › ijerph-1330776-supplementary.pdf]

Supplemental File

To Vaccinate or Not to Vaccinate—This Is the Question among Swiss University Students

Julia Dratva [1,2,*,†], Aylin Wagner [1,†], Annina Zysset [1] and Thomas Volken [1]

[1] Institute of Health Sciences, ZHAW Zurich University of Applied Sciences, Katharina-Sulzer-Platz 9,
8401 Winterthur, Switzerland; aylin.wagner@zhaw.ch (A.W.); annina.zysset@zhaw.ch (A.Z.); thomas.volken@zhaw.ch (T.V.)

[2] Medical Faculty, University of Basel, Klingelbergstrasse 61, 4056 Basel, Switzerland

* Correspondence: julia.dratva@zhaw.ch

† Authors contributed in equal shares (shared first authorship).

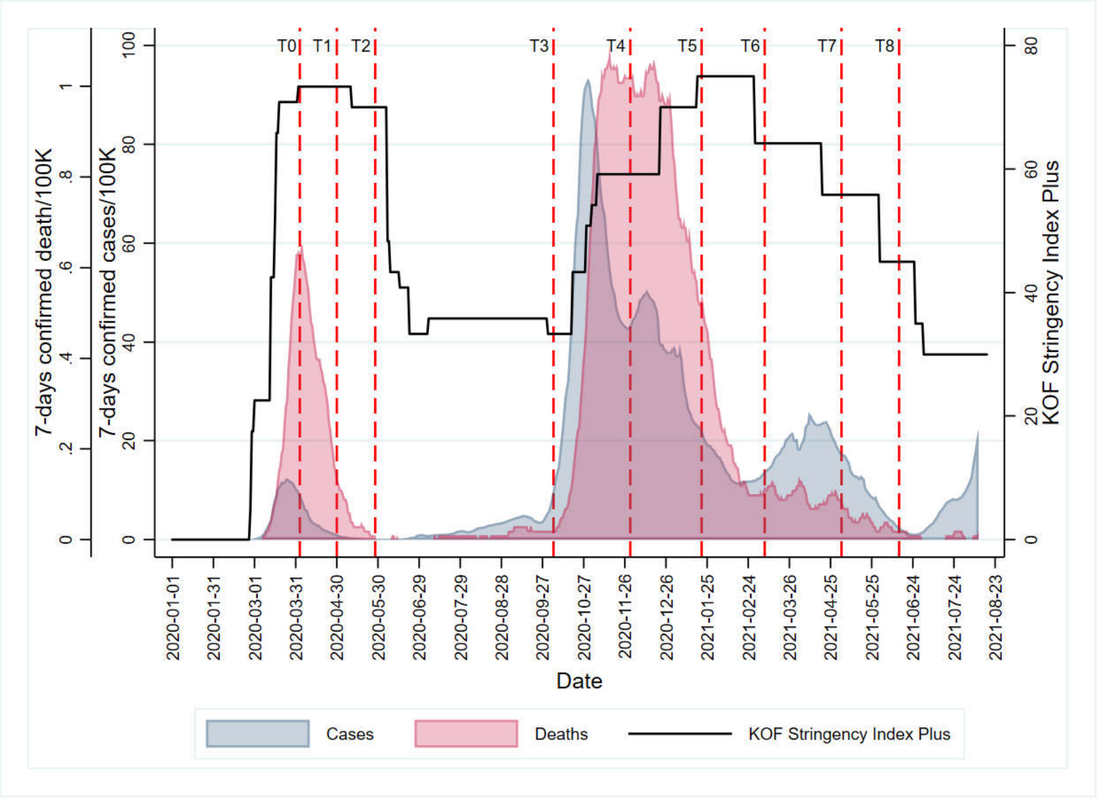

Figure S1. Epidemiological data and KOF Stringency Index over the duration of the HES-C study.

Legend:

T0 – T8 correspond to wave 1 – wave 9 of the HES-C study;

KOF Stringency Index records the stringency of COVID-19 containment measures in Switzerland [1].

Table S1. COVID-19 vaccination intention – Unadjusted ordinal logistic regression model

	Coefficient	95% CI
/Cut1	4.13	2.89 - 5.36
/Cut2	6.44	5.17 - 7.71
/Cut3	7.96	6.66 - 9.26
/Cut4	9.23	7.91 - 10.55
/Cut5	11.12	9.77 - 12.48
Number of observations	1297	
Pseudo R-squared	0	
Log likelihood	-2234	
Outcome	Predicted probability	95% CI
1	0.08	0.06 - 0.09
2	0.14	0.12 - 0.16
3	0.14	0.13 - 0.16
4	0.15	0.13 - 0.17
5	0.24	0.22 - 0.26
6	0.25	0.23 - 0.27

95% CI = 95% confidence interval

Reference

1. Pleninger, R.; Streicher, S.; Sturm, J.-E. Do COVID-19 Containment Measures Work? Evidence from Switzerland. **2021**, 53 p., doi:10.3929/ETHZ-B-000493408.
